# Allele-specific analysis of cell fusion-mediated pluripotent reprograming reveals distinct and predictive susceptibilities of human X-linked genes to reactivation

**DOI:** 10.1186/s13059-016-1136-4

**Published:** 2017-01-25

**Authors:** Irene Cantone, Gopuraja Dharmalingam, Yi-Wah Chan, Anne-Celine Kohler, Boris Lenhard, Matthias Merkenschlager, Amanda G. Fisher

**Affiliations:** 1 Lymphocyte Development Group, MRC London Institute of Medical Sciences, Hammersmith Campus, Imperial College London, Du Cane Road, London, W12 0NN UK; 2 Bioinformatics and Computing facility, MRC London Institute of Medical Sciences, Imperial College, London, UK; 3 Computational Regulatory Genomics Group, MRC London Institute of Medical Sciences, Imperial College, London, UK

**Keywords:** Reprograming, X chromosome reactivation, Stochasticity

## Abstract

**Background:**

Inactivation of one X chromosome is established early in female mammalian development and can be reversed in vivo and in vitro when pluripotency factors are re-expressed. The extent of reactivation along the inactive X chromosome (Xi) and the determinants of locus susceptibility are, however, poorly understood. Here we use cell fusion-mediated pluripotent reprograming to study human Xi reactivation and allele-specific single nucleotide polymorphisms (SNPs) to identify reactivated loci.

**Results:**

We show that a subset of human Xi genes is rapidly reactivated upon re-expression of the pluripotency network. These genes lie within the most evolutionary recent segments of the human X chromosome that are depleted of LINE1 and enriched for SINE elements, predicted to impair *XIST* spreading. Interestingly, this cadre of genes displays stochastic Xi expression in human fibroblasts ahead of reprograming. This stochastic variability is evident between clones, by RNA-sequencing, and at the single-cell level, by RNA-FISH, and is not attributable to differences in repressive histone H3K9me3 or H3K27me3 levels. Treatment with the DNA demethylating agent 5-deoxy-azacytidine does not increase Xi expression ahead of reprograming, but instead reveals a second cadre of genes that only become susceptible to reactivation upon induction of pluripotency.

**Conclusions:**

Collectively, these data not only underscore the multiple pathways that contribute to maintaining silencing along the human Xi chromosome but also suggest that transcriptional stochasticity among human cells could be useful for predicting and engineering epigenetic strategies to achieve locus-specific or domain-specific human Xi gene reactivation.

**Electronic supplementary material:**

The online version of this article (doi:10.1186/s13059-016-1136-4) contains supplementary material, which is available to authorized users.

## Background

In mammals, one of the two female X chromosomes is randomly inactivated to compensate gene expression between males (XY) and females (XX) [1]. This process takes place in the pluripotent cells of the early embryo and is inherited thereafter through cell division. As a consequence, female tissues comprise a mosaic of cells with reciprocal X chromosome inactivation (XCI) patterns that have been clonally inherited through development [2].

Silencing along the inactive X chromosome (Xi) leads to the formation of a condensed nuclear compartment, which excludes RNA polymerase and is enriched of repressive chromatin modifications (reviewed in [3]). *Xist* long non-coding RNA triggers the formation of this compartment by binding along the Xi [4] and then recruiting chromatin modifiers such as polycomb repressive complexes [5, 6]. Following these early events, gene silencing is established and spreads along the Xi with different kinetics [7–9]. Not all the genes on the Xi are effectively silenced and some genes escape inactivation and instead remain transcriptionally active [10, 11]. The heterogeneity in kinetics and efficiency of silencing suggests that local genetic and epigenetic features might determine the susceptibility of X-linked loci to inactivation. This hypothesis is supported by recent studies showing that the pre-existing chromatin context favours the recruitment of different sets of silencing factors and influences the efficiency with which XCI is established and propagated [12, 13]. Whether the local gene context also affects the maintenance of silencing and, therefore, the susceptibility of gene loci to be reactivated has been more difficult to interrogate.

The cell type, age and developmental context might also influence locus susceptibility to silencing and reactivation. Supporting this idea, recent studies in different mouse cell-types and tissues showed that some Xi genes escape silencing in a tissue-specific manner [14]. Furthermore, in mouse it has been shown that some genes escape silencing at the onset of XCI [15], whereas others are initially silenced in the embryo and are then reactivated later in development [16]. As genes that escape XCI lack *Xist* coating [8] and repressive H3K27me3 and H3K9me3 histone modifications [17, 18], one explanation could be that they retain an accessible chromatin during XCI and therefore are susceptible to subsequent reactivation. Supporting this hypothesis, RNA polymerase II has been shown to bind a subset of Xi genes that are silenced in some cell lines but active in others, possibly marking genes permissive to transcription [19].

Dynamic studies of the global Xi status during cell fate reprograming offer an opportunity to dissect the influence of pre-existing chromatin and cellular environments on Xi gene reactivation. Towards this aim, we examined Xi-specific expression during pluripotent reprograming of human female fibroblasts. XCI in humans has some intrinsic advantages for studying the impact of gene context and cellular environment on silencing or transcription. First, the percentage of genes escaping silencing in human is higher than in mouse (15% versus 3–7% of mouse), with 10% of genes showing a variable XCI status (i.e. escape or are subject to silencing) in different females or tissues [20]. This suggests that spreading of XCI in human might be less efficient and has evolved independently from different mammalian species. Second, compartmentalisation of the human X chromosome based on evolutionary, genomic and chromatin features has revealed some important associations between locus context and probability of silencing or escape. For example, many genes escaping XCI are contained within the evolutionary recent X Added Region (XAR) enriched for Alu repeats and depleted of Long Interspersed Elements (LINE) L1 and H3K27me3/H3K9me3 repressive histone modifications [17, 18, 21–25]. These findings may implicate a role for DNA elements, chromatin features and chromosomal domains in the spreading of human XCI.

Pluripotent reprograming approaches allow reproducible studies of Xi reactivation [26] and offer the possibility of investigating the predisposition of different loci. Interestingly, several studies in human induced pluripotent stem cells (iPSCs) have shown that the Xi can be either fully or partially reactivated in different clones [27–29]. A comparative analysis of the X:autosome expression ratio in several iPSC lines suggested that telomeric chromosome regions distal from *XIST* locus are preferentially reactivated [28]. However, a general consensus about the susceptibility of genes to X chromosome reactivation (XCR) and their location has not been achieved, mainly due to the variable outcomes of human iPSC reprograming and a paucity of studies that directly assess the expression of Xi-specific alleles across the entire X chromosome. Here, we used RNA-sequencing (RNA-seq) and allele-specific analyses to investigate human XCR during pluripotent reprograming of single-cell derived female fibroblast clones. Because XCR is a late event in iPSC-reprograming, is unstable and varies depending on the combination of inducing pluripotency factors and culture conditions (reviewed in [30]), we have used an alternative model system in which human fibroblasts (hFs) are reprogrammed by cell-fusion with mouse embryonic stem cells (mESCs). We have previously shown that interspecies cell fusions rapidly induce human pluripotency genes, giving us an opportunity to access the earliest events and the kinetics of the transition from the somatic to the pluripotent state [31, 32]. In this study, we determined the expression of human Xi genes in isogenic female fibroblast clones with different active/inactive X chromosomes ahead of and after cell fusion-mediated reprograming. We showed that a subset of genes (10% of those identified) is rapidly and consistently reactivated upon the induction of the human pluripotency gene network. Importantly, this group of genes were also shown to display variable Xi-allelic expression among individual hFs and between different single-cell derived clones, highlighting an intrinsic susceptibility to be expressed from the Xi.

## Results

### SNP detection by RNA-seq of female fibroblast clones discriminate the Xi and Xa chromosomes

**Fig. 1:**
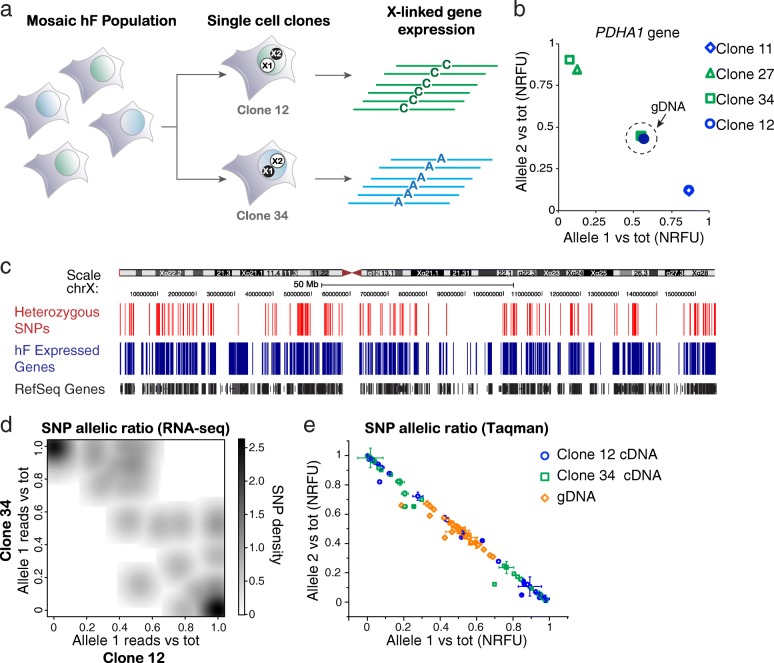
Allele-specific expression in single cell-derived clones discriminates the Xa and Xi. **a** *Scheme* illustrates the experimental design. Single cells that express/silence reciprocal X chromosomes (i.e. X_1_aX_2_i or X_1_iX_2_a) were isolated from a female (X_1_X_2_) hF line with balanced XCI (i.e. NHDF17914) and expanded to derive reciprocal isogenic clones (e.g. clone 12 and 34). Expression analysis of heterozygous SNPs within X-linked genes then allowed the active (Xa, *open white circle*) and inactive (Xi, *closed black circle*) X chromosomes in each clone to be distinguished. **b** *Plot* showing allele-specific expression of the X-linked gene *PDHA1* in four hF clones where the allelic ratio was determined by SNP-specific Taqman probes and reported as normalised relative fluorescent units (NRFU) vs. total (allele1 + allele2). Using a known heterozygous SNP (rs1042456), clones 11 and 12 (*blue diamond* and *circle*) are shown to express a distinct allele from clones 27 and 34 (*green triangle* and *square*) while both alleles were detected in genomic (gDNA) hF samples (*dashed circle*). **c** Heterozygous SNPs identified by RNA-seq (i.e. 379 SNPs by strategy 1 in Additional file 1: Figure S1) are represented as *red lines* along the X-chromosome ideogram. Genes expressed in fibroblasts (FPKM >25th percentile) and reference genes (USCS Genome browser) are shown in *blue* and *black*, respectively. **d** *Density plot* represents the overall allelic expression in clone 12 and 34 obtained by RNA-seq ahead of data modelling. For each of the 379 heterozygous SNPs, the ratio between the SNP-reads overlapping allele 1 (i.e. the ones matching the SNP reported in the Reference genome sequence) vs. the total is shown, where the intensity of grey scale represents the density of SNPs with a certain allelic ratio. **e** *Plot* shows allelic ratios of 52 heterozygous SNPs that were identified by RNA-seq. Data represent the average of at least two independent samples for each clone and are plotted as normalised relative fluorescent units (NRFU) of each allele vs. the total. *Error bars* indicate standard errors of mean (SEM)

In order to investigate the global expression from the active (Xa) X chromosomes and Xi, we used RNA-seq and single-cell derived clones. Clonal selection allows homogeneous cell lines to be derived with either one (X_1_) or the other (X_2_) inactive chromosome (X_1_aX_2_i or X_1_iX_2_a, as illustrated in Fig. 1a). To discriminate between the two X chromosomes, we used a hF cell line with a known single nucleotide polymorphism (SNP) in the X-linked *PDHA1* gene [33] and immortalised cells by exogenously expressing *TERT* in order to derive single-cell clones. Analysis of *PDHA1* gene expression using SNP-specific Taqman probes confirmed reciprocal Xa/Xi patterns within four isogenic hF clones (X_1_aX_2_i in clones 11 and 12, X_1_iX_2_a in clones 27 and 34, Fig. 1b). Extending the same principle to all X-linked genes, we reasoned that we could identify heterozygous SNPs by looking for positions along the X chromosome at which a different nucleotide was expressed in reciprocal clones (Strategy 1, illustrated in Additional file 1: Figure S1). This relies on the assumption that alleles on the Xi are either not expressed or expressed at lower level than their counterparts on the Xa [10]. Briefly, heterozygous SNPs were found by mapping RNA-seq reads, identifying the ‘observed base’ (i.e. the most abundant base within the uniquely mapped reads) at each genomic position on the X and selecting those positions at which a different base was observed between reciprocal clones (Additional file 1: Figure S1). We obtained a dataset including 379 SNPs within 183 genes along the X chromosome (Fig. 1c and Additional file 2: Table S1), which represent approximately 25% of X-linked genes expressed by the hFs. In total, 96% of these putative heterozygous SNPs were reported in the human SNP database [34] and 56 SNPs were validated on genomic DNA of both clones (using Sanger sequencing or Taqman probes; Fig. 1e and Additional file 2: Table S1). An alternative strategy was also used to find heterozygous SNPs by merging RNA-seq data from reciprocal clones to mimic genomic DNA (Strategy 2, Additional file 1: Figure S1), as previously reported [35]. This identified a total of 355 SNPs, of which 354 overlapped with those identified previously (Strategy 1) validating this dataset. In addition, comparisons of SNP-specific expression using RNA-seq (Fig. 1d) or Taqman (Fig. 1e) of hF clones 12 and 34 confirmed that most X-linked genes were transcribed only from one X chromosome and that these clones expressed opposite haplotypes along the entire chromosome.

To determine allele-specific expression based on genes (rather than single SNPs) directly, we summed SNP-specific reads within the same gene and modelled Xi expression using a beta-binomial distribution, as previously described [36]. Briefly, data obtained from two replicates of each clone were merged and the gene probability of being inactivated (τ_i0_, see Table 1) was modelled based on the proportion of Xi expression versus total, the number of Xi-specific reads and the base quality scores within these reads. Previous studies considered genes as expressed from the Xi based on an empirical threshold of 10% of the Xa [10] and classified such genes as ‘escaping XCI’ from the analysis of mosaic cell populations with unbalanced XCI [25, 37]. Beta-binomial modelling allowed us instead to assess the statistical significance within our hF clones and showed that approximately 15% of genes were significantly expressed from the Xi in at least one of the two somatic clones examined (21 out of 145 genes with SNP-reads ≥20 in both clones), consistent with previous reports [10]. A good agreement was found between our data and two previous analyses of human X chromosome inactivation based on allelic imbalance in human female cell lines with skewed XCI [25, 37]. As shown in Table 1, we identified 21 genes significantly transcribed from the Xi (τ_i0_ < 0.5) of which 16 were previously reported as escaping XCI [25, 37].

**Table 1:** Genes with significant Xi expression in hF clone 12 or 34

Gene	Clone 12					Clone 34				
	Xa reads	Xi reads	Total reads	% Xi	τi0	Xa reads	Xi reads	Total reads	% Xi	τi0
*ARSD*	238	236	474	49.8	0	284	14	298	4.7	1.52E-06
*CA5B*	298	133	431	30.9	0	255	42	297	14.1	0
*CDK16*	4232	525	4757	11.0	0	3647	428	4075	10.5	0
*CENPI* ^a^	167	6	173	3.5	0.0113	114	8	122	6.6	1.11E-05
*CXorf40A*	1341	3	1344	0.2	0.9975	726	191	917	20.8	0
*GEMIN8*	61	46	107	43.0	0	83	25	108	23.1	0
*GYG2*	73	31	104	29.8	0	27	0	27	0.0	0.9834
*HCCS* ^a^	23	71	94	75.5	0	221	0	221	0.0	0.9951
*MED14*	441	34	475	7.2	0	268	15	283	5.3	1.73E-07
*MXRA5*	9706	97	9803	1.0	0.0194	6020	827	6847	12.1	0
*PLCXD1*	34	22	56	39.3	0	16	35	51	68.6	0
*PNPLA4*	106	19	125	15.2	0	108	2	110	1.8	0.6737
*PRKX*	298	2	300	0.7	0.9898	62	6	68	8.8	2.04E-05
*RIBC1*	18	6	24	25.0	1.73E-08	22	0	22	0.0	0.9812
*RP11-1148 L6.5*	835	0	835	0.0	0.9998	137	5	142	3.5	0.0153
*RP11-706O15.1*	564	24	588	4.1	2.18E-08	392	5	397	1.3	0.5506
*SYAP1*	45	16	61	26.2	0	44	6	50	12.0	6.16E-06
*TMSB4X*	31,358	660	32,018	2.1	4.15E-06	32,145	9	32,154	0.0	0.9873
*UXT*	48	26	74	35.1	0	30	33	63	52.4	0
*WASH6P*	13	11	24	45.8	0	12	10	22	45.5	0
*XG*	4868	4337	9205	47.1	0	3878	561	4439	12.6	0

^a^Genes with discordant XCI status in previous studies

For 76 genes containing more than one SNP, we also calculated the probability of Xi expression for each single SNP position (Additional file 2: Table S1). This analysis showed that more than 98% of SNPs had concordant Xi-allelic expression within the same gene. The few SNPs that showed discordant Xi expression probability with others along the same gene (7 and 5 SNPs out of 328 total in clones 12 and 34, respectively) had low Xi reads and did not change the probability of Xi expression when summed up. Overall, these data confirm that RNA-seq allowed robust identification of heterozygous SNPs along the Xa and Xi and thereby to discriminate Xi genes that expressed or silenced.

### Cell-fusion between female hF clones and mESCs induces expression of the human pluripotency network

**Fig. 2:**
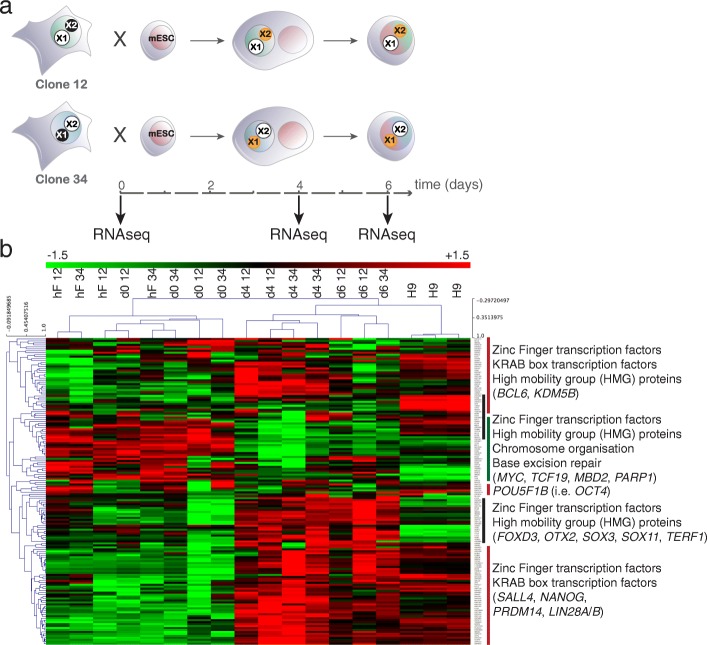
hF clones are reprogrammed towards an embryonic pluripotent state by fusion with mouse ESCs. **a** Strategy used for examining Xi-gene reactivation during pluripotent reprograming of female hF clones. Isogenic clones with opposite XCI patterns (i.e. X_1_aX_2_i and X_1_iX_2_a) were fused with mESCs and RNA-sequencing was performed at 0, 4 and 6 days after fusion. **b** Hierarchical clustering of transcription factor genes that are part of the human pluripotency-associated gene regulatory network [43] in hESC (i.e. H9), hF clones (i.e. clone 12 and 34) and at 0, 4 and 6 days after mESC-fusion. Displayed values correspond to the expression level (rlog, regularised logarithmic transformation) that has been scaled by the mean expression of each gene across all samples. Expression levels were computed using RNA-seq reads that uniquely aligned to the human reference genome, but not to mouse. *Sidebars* highlight major clusters and are annotated with gene ontology. *Red* and *green bars* mark genes that are, respectively, upregulated or downregulated regulated upon cell fusion-mediated reprograming. *Black bars* mark clusters that are upregulated upon cell fusion but not in hESCs, or vice versa

To identify X-linked genes that were susceptible to reactivation during the acquisition of a pluripotent state, we then reprogrammed hF clones via cell fusion with mESCs and performed RNA-seq at 0, 4 and 6 days after fusion (Fig. 2a). Global RNA-seq analysis of the human-specific reads (approximately >95% and 50% of total reads from fibroblast and hybrid samples, respectively; Additional file 3: Table S2) showed significant upregulation of developmental genes including signalling pathways and cell cycle genes that specifically sustain human pluripotency (e.g. TGF-beta, insulin/IGF and cyclins D1-D3) [38–40] and downregulation of tissue-specific genes including those encoding fibroblast-associated functions (e.g. extracellular matrix organisation and cell adhesion) (Additional file 1: Figure S2A). Furthermore, genes differentially expressed upon cell-fusion were enriched for NANOG and OCT4 direct targets [41] (Additional file 1: Figure S2B) consistent with reprograming of hFs towards a pluripotent-like state [42]. To investigate the extent of cell fusion mediated-reprograming, we assessed the expression of transcription factors that have been described as part of the human embryonic stem cell (hESC)-specific gene regulatory network by a recent systems biology approach (i.e. CellNet, [43]). Unsupervised clustering showed that, after fusion, hFxmESC cells had an expression profile that is more similar to hESC (control H9) than hFs (Fig. 2b). Interestingly, while the expression of some pluripotency factor genes (*SALL4*, *NANOG*, *PRDM14* and *LIN28*, lower cluster) increased at day 4 and day 6 to levels that were similar to those detected in hESCs, other factors including *OCT4* (*POU5F1*) were more variable. Transcription of factors involved in genome organisation and DNA repair appeared less abundant in hFxmESC and hESC samples than in hF clones (Fig. 2b). These data were consistent with cell fusion-mediated reprograming inducing a generalised reactivation of the human-specific pluripotency network in hF clones as well as changes in transcriptional profile that reflect alterations in cellular potential [32]. Of note, clustering analysis showed that a subset of pluripotency-associated transcription factors was induced to higher levels than found in control hESCs (clusters highlighted by black lines). Close inspection revealed that these genes characterise naïve hESCs (Additional file 1: Figure S2C), which are thought to resemble pluripotent cells of the human blastocyst [44]. This suggests that cell fusion-mediated reprograming induces a spectrum of genes that may encompass both the naïve and the primed pluripotent state.

### X chromosome-wide analysis of allelic expression reveals rapid reactivation of a subset of Xi genes during human pluripotent reprograming

**Fig. 3:**
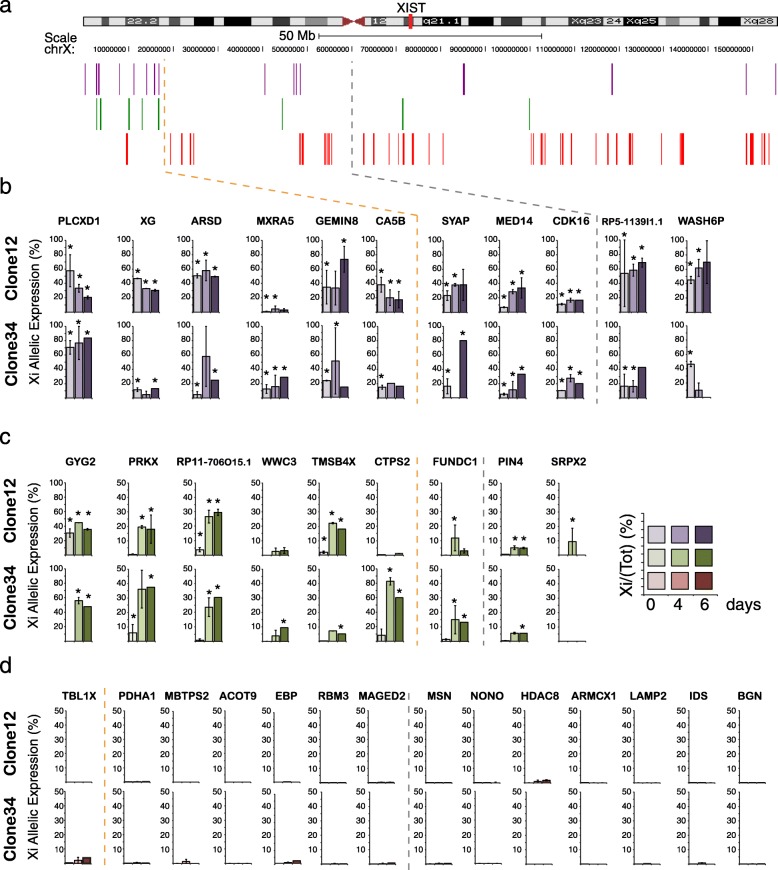
Allele-specific RNA-seq reveals differential sensitivity of Xi genes to reprograming-mediated reactivation. **a** *Summary scheme* showing the relative positions of human Xi genes that are significantly expressed before and after reprogramming in clones 12 or 34 (*purple*), are reactivated (*green*) or that remain inactive (*red*) upon reprograming; analysis based on allele-specific RNA-seq of reciprocal hF clones 12 and 34 (i.e. X_1_aX_2_i or X_1_iX_2_a; see Fig. 1a). **b**–**d** *Histogram plots* show the % of minor allele (Xi) vs. total SNP-reads per gene, as assessed by RNA-seq analysis (mean ± SEM of two biological replicates for each clone). SNPs with the lowest number of overlapping reads in hF clone 12 were considered to be Xi alleles (and to be Xa alleles in reciprocal clone 34). *Asterisks* (*) mark samples showing significant expression from Xi allele in replicate merged data (probability of inactivation τ_i0_ < 0.05, β-binomial statistics). Complete dataset analysis is reported in Table S3

**Fig. 4:**
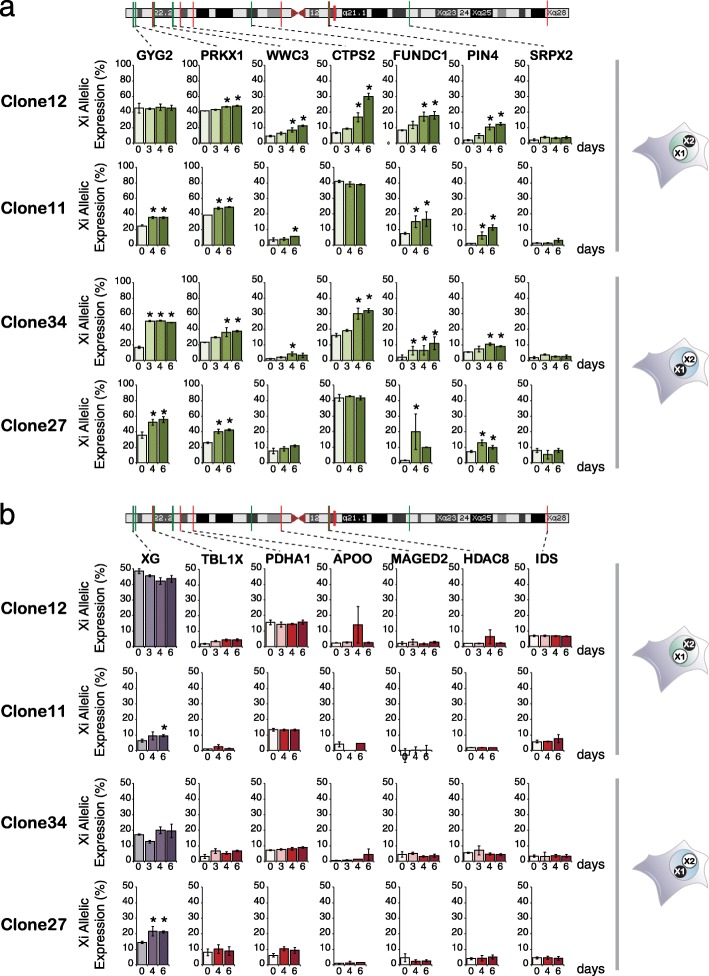
Differential sensitivity of Xi genes to reprograming-mediated reactivation is validated by SNP-specific Taqman probes and extended to four distinct hF clones. **a**, **b** *Histogram plots* show Xi-specific allelic expression of human X-linked genes in hFxmESC at 0, 3, 4 and 6 days after fusion. Genes that accordingly to RNA-seq are reactivated (*green*) or remain inactive (*red*) upon reprograming or that already have significant Xi expression in clones 12 or 34 ahead of reprogramming (*purple*) are shown and their locus positions are highlighted along the chromosome ideogram as vertical lines. Allelic expression was measured by Taqman SNP-specific probes and data represented as a percentage of the Normalised Relative Fluorescence Units (NRFU) for the Xi probe vs. the total (Xi + Xa + background in no template control). Data represent the average of at least five independent experiments for each clone ± SEM; day 3 samples were collected only in two independent experiments. *Asterisks* (*) mark significant differences vs. day 0 (*p* <0.05, two-sided t-test)

The extent of Xi gene reactivation induced by pluripotent reprograming was examined by RNA-seq of reciprocal hF clones before and at 0, 4 and 6 days after fusion with mESCs (two independent biological replicates per clone, Fig. 3). For each sample, we considered only those genes with 20 or more allele-specific reads in at least one sample obtained before (i.e. hF or day 0) and after reprograming (i.e. day 4 or day 6) and estimated the probability that a gene is inactivated (87 genes, Additional file 4: Table S3). Based on these results, we classed Xi genes into three distinct categories (Fig. 3a). We identified 16 genes that were expressed both before and after reprograming (Fig. 3b, purple), nine genes that showed significant Xi expression only after reprograming (i.e. reactivated, Fig. 3c, green) and 62 genes that remained silenced, and indeed subject to XCI, after reprograming (Fig. 3d, red). These results highlight substantial differences in the sensitivity of loci on the human Xi to reprograming-mediated reactivation. To confirm and extend this analysis, we used allele-specific Taqman probes to determine Xi gene expression in response to reprograming in each of the four hF clones described previously (i.e. clones 11, 12, 27 and 34). We observed significant re-expression upon reprograming of many reactivation-sensitive candidate genes (6 out of 7, Fig. 4a green) but not of reactivation-resistant candidates (0 of 6, Fig. 4b red). Although these data broadly confirmed the results of RNA-seq, closer inspection revealed a surprising level of stochastic expression among reactivation-sensitive Xi genes. In particular, several Xi genes that appeared to be induced upon reprograming in specific clones (e.g. *GYG2* in clone 34, 11 and 27; *CTPS2* in clone 12 and 34) were already expressed in others ahead of reprogramming (e.g. *GYG2* in clone 12, *CTPS2* in clone 11 and 27). This raised the possibility that the genes displaying variable Xi expression among human fibroblast clones might be particularly responsive to reprograming-mediated reactivation and vice versa.

### Local chromatin context and reprograming-mediated human Xi gene reactivation

An alignment along the linear DNA sequence of the human X chromosome revealed that most of the reprograming-reactivated gene loci clustered on the short Xp arm within topologically associated domains (TADs) that are enriched for genes escaping XCI in somatic cells (Additional file 1: Figure S3A) [45–47]. Genes neighbouring other regions prone to escape, for example in the distal Xq arm, were not particularly sensitive to reactivation upon reprograming. Furthermore, regions adjacent to the *XIST* locus or close to the centromere were not immune to reactivation, as exemplified by *PIN4*. Instead, reactivated genes appeared to be preferentially located in evolutionarily recent regions (strata S3–S5 illustrated in Additional file 1: Figure S3A) that were enriched for Alu repeats and depleted of LINE L1 elements [48, 49].

Since reprograming induced upregulation of human pluripotency-associated factors including OCT4 and NANOG, we asked whether the sensitivity of target genes to reactivation might correlate with either the extent of gene induction or the binding of such factors. As anticipated, reactivated genes were upregulated upon reprograming (Additional file 1: Figure S3B, green), while genes that remained active (purple) or inactive (red) throughout reprogramming showed no change in expression. Further analysis of Xa-specific and Xi-specific reads showed that, although induction reflected both re-expression of silent Xi alleles and increased Xa transcription (Additional file 1: Figure S3C), these changes were unlikely to be directly mediated by OCT4 or NANOG as these factors bound similarly to reactivated, active and inactive genes in hESCs (Additional file 1: Figure S3D).

To investigate whether the underlying chromatin state influence locus susceptibility to reactivation, we analysed H3K27me3 and H3K9me3 chromatin immunoprecipitation sequencing (ChIP-seq) from published datasets of immortalised human fibroblasts [50] (Additional file 1: Figure S3A and E). These repressive histone modifications are preferentially enriched along distinct regions of the Xi (Additional file 1: Figure S3A), which were previously shown to form two spatially distinct compartments [50, 51]. Xi genes that were reactivated upon cell fusion-mediated reprograming were preferentially localised to H3K27me3-enriched macro-domains (Additional file 1: Figure S3A, dashed rectangles). At the level of individual genes, however, the average enrichment of H3K27me3 or H3K9me3 along the gene body did not show significant differences between candidate reactivated and inactive genes (Additional file 1: Figure S3E). As the correlation between histone modifications and gene reactivation might be masked where values for Xa and Xi were combined, rather than separately assayed, we also performed ChIP for H3K27me3 and H3K9me3 in reciprocal hF clones 12 and 34 and analysed the ChIP by allele-specific probes (Additional file 1: Figure S4A and B). This analysis of candidates occupying three distinct TADs showed that the Xi-specific alleles of reactivated genes generally had low H3K9me3 along the gene body, but variable levels of H3K27me3, a profile that resembled genes that were already significantly expressed from the Xi before reprogramming, such as *XG* in clone12 and 34. Detailed comparisons of reactivation-sensitive (green) and inactive loci (red) indicated that H3K27me3 and H3K9me3 levels did not however determine the sensitivity of loci to reactivation-mediated reprograming (Additional file 1: Figure S4C).

### Human Xi genes that are susceptible to reprograming-mediated reactivation show stochastic expression in human fibroblasts

**Fig. 5:**
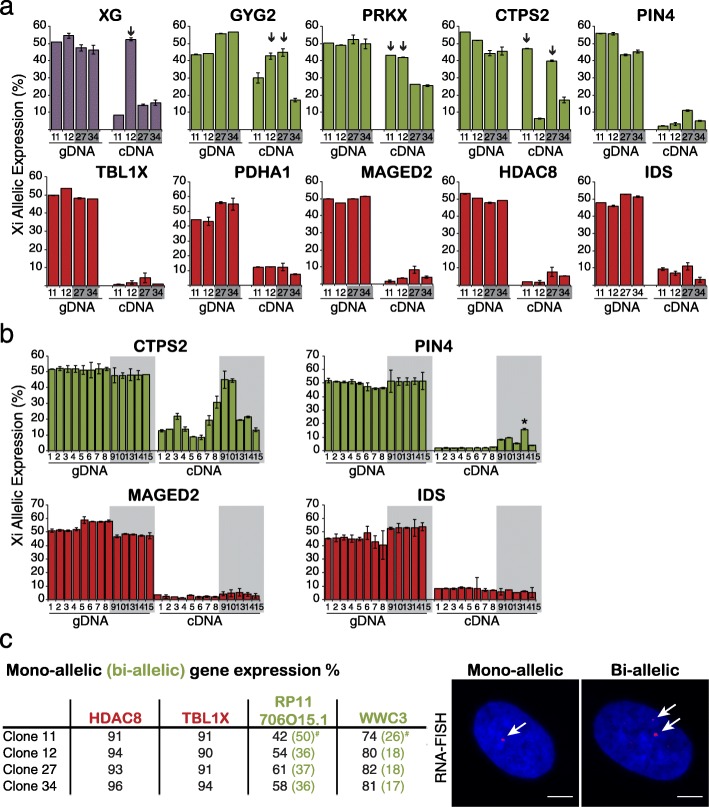
Variable allelic expression of Xi genes in hFs predicts their susceptibility to reprograming-mediated reactivation. **a** *Histograms* show Xi-allelic expression (as a percentage of total) of 10 X-linked genes in hF clones 11 and 12 and reciprocal clones 27 and 34 (*grey*), where gDNA provides controls. Allelic expression was measured using Taqman probes as NRFU of the Xi-specific probe vs. the total; data for each hF clone represent the average of at least three independent cultures. *Arrows* indicate significant differences in Xi expression (*p* <0.05, two-sided t-test) as compared with data from other clones. Genes that accordingly to RNA-seq are stably expressed (*purple*), reactivated (*green*) or remain inactive (*red*) across reprograming in clones 12 or 34 are shown. **b** Xi-allelic expression of *CTPS2*, *PIN4* (reactivated genes), *MAGED2* and *IDS* (inactive) in 13 additional hF clones. Analysis was performed as in (**a**). *Asterisk* (*) marks clones with Xi expression significantly different from the others (*p* <0.05, Grubbs test). **c** Mono-allelic and bi-allelic expression of X-linked genes in hFs was determined using RNA-FISH (exemplified in the *images* shown to the *right*) where punctate signals (*red*, *arrowed*) represent transcribed X-linked gene loci. At least 200 nuclei containing either a single punctate signal (mono-allelic) or two separate signals (bi-allelic) were scored for each hF clone and the percentage of total cells showing mono-allelic or bi-allelic (in *brackets*) patterns is shown. For *HDAC8* and *TBL1X* >90% of the analysed cells showed mono-allelic expression. # indicates samples with higher bi-allelic expression than others (RNA-FISH) where significant Xi expression was also detected (RNA-seq). DAPI (*blue*) stains nuclei and *scale bars* indicate 5 μm

Reprograming assays indicate that X-linked genes have distinct sensitivities to reactivation. In addition, the expression of some reactivation-sensitive genes varied considerably between different hF clones isolated from the same female (Fig. 4a, green). To investigate the significance of this observation we compared the allelic expression ratios of four genes identified by RNA-seq as reactivation-sensitive (green) with five reactivation-resistant (red) candidates in cDNA samples from each hF clone, where genomic DNA (gDNA) was used as an internal control (Fig. 5a). Expression of the ‘reactivation-sensitive’ candidates differed considerably between the four hF clones. For example, *CTPS2* was expressed at similar levels from the Xi and Xa in clones 11 and 27 (Xi expression was approximately 50% of the total in both genomic DNA and cDNA, arrows in Fig. 5a), whereas in clones 12 and 34, Xi expression was significantly lower (less than 20% of the total). Variable allelic expression was a feature of each of the loci that showed reactivation upon reprograming (*GYG2*, *PRKX* and *CTPS2*) but not those genes that were refractory to reactivation (*TBL1X*, *PDHA1*, *MAGED2*, *HDAC8* and *IDS*). Likewise, among a panel of 13 independently isolated hF clones that originated from the same donor, variable allelic expression was selective for the reactivation-sensitive candidates (*CTPS2* and *PIN4*, Fig. 5b, green). Of note, *XG* that was significantly expressed from the Xi across reprogramming in clones 12 and 34 showed instead variable Xi expression in additional clones (Fig. 5a, purple and Additional file 2: Table S1) and was indeed susceptible to reactivation upon reprograming of these clones (Fig. 4b). These data suggest genes that are sensitive to reprograming-mediated reactivation may be stochastically expressed in hFs. To verify this at the level of single cells rather than clonal populations, we performed RNA-FISH to examine mono-allelic and bi-allelic expression of human X-linked genes in individual fibroblast nuclei. As shown in Fig. 5c, genes such as *HDAC8* and *TBLX1* that resisted reprograming-mediated reactivation, showed mono-allelic expression in the majority (93–96% and 90–94%) of cells examined from multiple hF clones. In contrast, we observed bi-allelic expression of the reactivation-sensitive candidate genes *WWC3* and *RP11706015.1* in a proportion of hFs (17–26% and 36–50%, respectively) with substantially higher percentages in one of the four analysed clones (i.e. clone 11). These data supported the view that this subset of human X-linked genes is particularly vulnerable to ‘stochastic’ transcription in fibroblasts, a property that may well reflect intrinsic differences in mechanisms that regulate gene expression in this somatic cell type. In support of this claim, RNA-seq analysis showed that variability in Xi expression between isolated fibroblast clones was common among X-linked genes identified as being sensitive to reprograming-induced reactivation (6/9 genes with significant Xi expression in at least one clone; τ_i0_ ≤ 0.05) while among genes that resisted reactivation, variability was instead rare (3/62 genes analysed) (Additional file 5: Table S4).

### Xi genes that are sensitive to DNA demethylation are revealed through reprograming

Previous studies have shown that DNA demethylation triggers the sporadic reactivation of Xi alleles in somatic cells [52, 53] and is required for reprograming-induced Xi reactivation [29, 54]. We therefore checked whether global DNA demethylation induced by 5-deoxy-azacytidine (5azaC; Additional file 1: Figure S5A) would increase Xi transcription at active, reactivated or stably inactive genes. Although 5azaC treatment was able to significantly increase Xi expression ahead of reprograming (from 18% to 28%) at *XG* locus in clone 34 (Additional file 1: Figure S5B), we did not observe significant changes in Xi expression at any of the candidate reactivation-sensitive (*GYG2*, *PRKX*, *CTPS2*, *FUNDC1*, *PIN4* and *SRPX2*) or reactivation-resistant genes (*TBL1X*, *PDHA1*, *APOO*, *MAGED2*, *HDAC8* and *IDS*) tested. This suggested that DNA methylation was unlikely to be the cause of the stochastic Xi re-expression in hF clones.

**Fig. 6:**
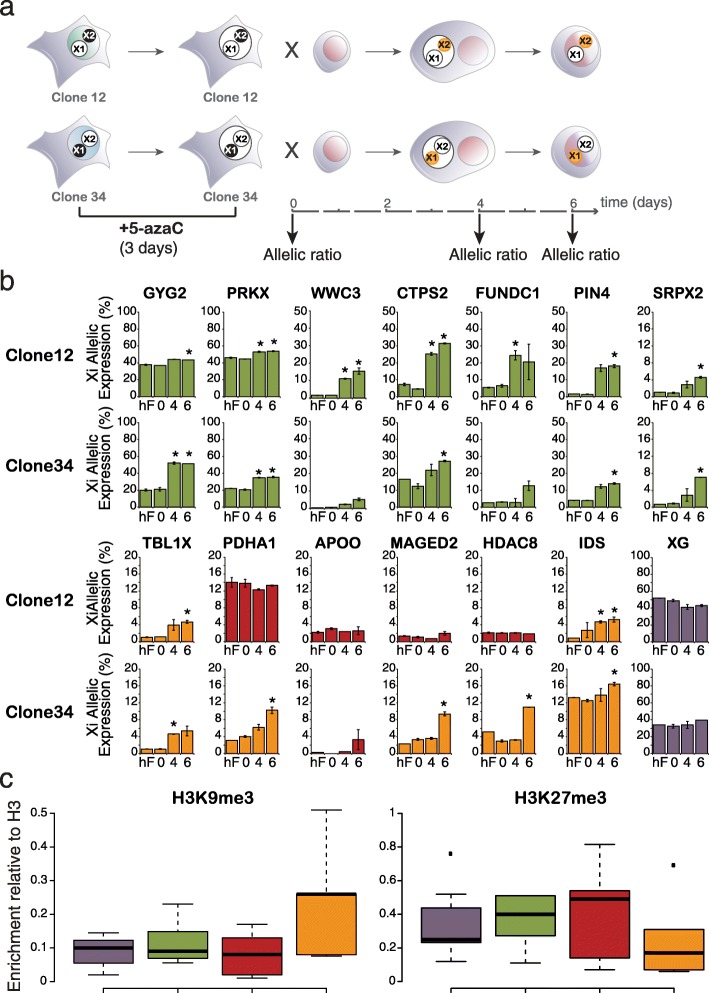
DNA demethylation induces Xi reactivation upon reprograming at loci within high H3K9me3 and low H3K27me3 domains. **a** *Scheme* illustrates the experimental outline. hF clones were cultured in the presence of 5-azaC for three days before fusion with mESCs. 5-azaC was removed from cultures at fusion and allelic expression of X-linked genes was analysed 0, 4 and 6 days post fusion. **b** *Histogram plots* show Xi-allelic expression during reprograming upon 5azaC pre-treatment. Inactive alleles with a significant Xi induction upon 5azaC pre-treatment and cell fusion-mediated reprograming are shown in *orange*, whereas refractory alleles are shown in *red. Asterisks* (*) mark significant changes vs. day 0 (*p* ≤0.05, two-sided t-test). Allelic expression was measured by Taqman probes as NRFU of the Xi-specific probe versus the total. Data represent the average of two independent experiments ± SEM for each clone. **c** *Box plots* show Xi-specific enrichment of H3K9me3 and H3K27me3 in clones 12 and 34. Xi enrichment has been detected by using SNP-specific Taqman probes and normalising Ct values to H3 ChIP and input DNA. ChIP was performed in at least two independent experiments for each clone

As both DNA demethylation and *XIST* loss were suggested to be required for X chromosome reactivation in mouse [54, 55], we reasoned that 5azaC might enhance reactivation upon reprograming. We therefore treated hF clones for three days before mESC-fusion and analysed Xi-specific expression in hFxmESC cells at 0, 4 and 6 days as outlined in Fig. 6a. Xi expression of reactivated genes was induced at similar levels and with the same kinetics as in the absence of 5azaC (Fig. 6b, green), consistent with the susceptibility of these genes to reactivation being independent of DNA methylation. However, a different subset of genes was consistently reactivated upon 5azaC pre-treatment and cell fusion-mediated reprograming (Fig. 6b, yellow) including candidates such as *TBLX1* and *HDAC8* that had previously resisted reactivation. Longer 5azaC treatment provided moderate increases in Xi-expression levels upon reprograming (Additional file 1: Figure S5C). As several genes were refractory to 5azaC-reprograming-mediated reactivation in one hF clone but induced in another (*PDHA1*, *MAGED2* and *HDAC8* in clones 12 vs. 34, Fig. 6b) we asked whether this reflected differences in underlying H3K27me3 or H3K9me3 levels. Among the candidates examined, sensitivity to 5azaC pre-treatment correlated with high levels of H3K9me3 and low levels of H3K27me3 (orange), as compared with genes previously characterised as active (purple), reactivated (green) or that were refractory to reactivation across reprograming (red, Fig. 6c).

Our results show that loci along the human X chromosome have distinct susceptibilities to reprograming-mediated reactivation that reflects both their genomic context and the local chromatin environment that is resident (or induced) within somatic cells. Among the 87 human X genes sampled here, we have identified nine that are reactivated upon reprograming (10%) and 62 that remain inactive (71%). Seven genes within the inactive cohort (11%) were shown to be DNA methylation-sensitive. In addition, genes identified as being particularly prone to reprograming-mediated XCR also showed evidence of variable Xi expression within individual hFs and hF clones, suggesting that these two processes are mechanistically related.

## Discussion

Our study set out to examine human X chromosome reactivation directly during pluripotent reprograming of somatic cells using allele-specific RNA-seq approaches. We have examined 87 X-linked genes and provide the first direct and quantitative evidence of Xi transcription in several hF clones before, and immediately after, the induction of pluripotency. Previous iPS studies have generally used indirect measures of XCR, such as X:autosome expression ratios [28, 56] or loss of DNA methylation [29, 57], to gauge Xi gene expression. Here, we have instead used allele-specific RNA-seq to discriminate transcripts originating from the Xa and Xi and to directly quantify their expression during reprograming. We have applied statistical modelling to directly assess significant Xi expression rather than relying on empirical definitions of XCI escape as being greater than 10% of Xa expression. Recent studies in mouse cells and tissues have shown that allele-specific RNA-seq detects a wider range of Xi expression and allows genes that escape XCI to be estimated with a higher level of sensitivity than previous techniques [14, 36]. Using a similar approach, we show selective reactivation of genes along the human Xi upon induction of the human pluripotency network. Due to the epigenetic instability of the Xi in human pluripotent cells [27, 58], and differences in the reprograming routes of iPSC versus cell–cell fusions, direct comparisons between different approaches is problematic. However, one of the genes described in our analysis, *GYG2*, has been shown to reactivate during early iPSC reprograming [59]. Recent reports also show that Xi genes that are reactivated at early iPSC stages can undergo subsequent de novo inactivation, probably due to inappropriate culture conditions [59, 60]. Interestingly, some genes that have been shown to be reactivated at late iPSC stages, including *PDHA1* and *IDS*, are reactivated by cell fusion-mediated reprograming after 5azaC pre-treatment of somatic clones. This suggests that fusion with mESCs promotes a different (and potentially faster) reprograming route than iPSCs and supports claims that DNA demethylation is required for the reactivation of a subset of human Xi genes during pluripotent reprograming [29].

From our RNA-seq analysis, we estimate that Xi reactivation occurs in about 10% of human X-linked genes. This selectivity could reflect the mechanism by which silencing is propagated along the X chromosome and, therefore, underlying genetic and epigenetic differences. It was originally proposed that silencing spreads linearly from the *XIST* locus into adjacent and more distal DNA, aided by specific repetitive sequences [61]. However, recent studies in mouse showed that *Xist* RNA first binds to loci that are in close spatial proximity to its locus [8, 9] and then propagates to inactivated genes, suggesting that ‘spatial’ spreading may underpin XCI. Here, we show that reactivation of genes along the human Xi is not limited to telomeric regions distal to the *XIST* locus, as previously suggested [28]. We observe instead that several reactivated genes localise within TADs enriched of genes constitutively escaping XCI (i.e. *GYG2*, *PRKX*, *RP11-706O15.1* and *CTPS2*). Recent studies showed that chromatin domains along the Xa and Xi have a distinct spatial organisation [62] with TAD-like structures that retain DNA accessibility being present along the Xi only around genes escaping XCI [47]. As loci of escaping Xi genes have been shown to interact within each other [63] spatially segregating from inactive loci [64, 65], our data support the hypothesis that three-dimensional chromatin organisation might influence gene susceptibility to expression. Interestingly, reprograming-reactivated genes within the same TAD of loci that remained inactive were enriched in SINEs and depleted in L1 LINEs, confirming that repeat sequences also contribute to the spread of silencing along the X chromosome [20].

Since two spatially distinct heterochromatin compartments can be distinguished along the human Xi based on the preferential enrichment of H3K9me3 or H3K27me3 [50, 51], we also investigated whether these chromatin features influence the predisposition of genes to reactivation. Analysis of published ChIP-seq data showed that reactivated genes preferentially localised within macro-domains enriched in H3K27me3 and depleted in H3K9me3 (dashed squares in Additional file 1: Figure S3A). Allele-specific ChIP analysis in our hF clones also confirmed that Xi loci of reactivated genes have low levels of H3K9me3 ahead of reprograming, consistent with previous reports that H3K9me3 can act as a barrier for reprograming [66, 67]. Interestingly, a recent study has shown that *XIST* RNA mainly associates with H3K27me3-enriched domains in hFs, whereas the heterochromatin protein HP1α co-localises with H3K9me3 domains [50]. Furthermore, loss of *XIST* RNA in hESC lines that have already undergone XCI leads to the reactivation of genes within H3K27me3 domains [58]. Collectively, these data suggest that silencing of distinct loci along the human Xi might be maintained by partially overlapping XIST-dependent and XIST-independent mechanisms, and that genes within H3K27me3 domains (and depleted in H3K9me3) might be more susceptible to reactivation in a pluripotent cellular environment. Consistent with this hypothesis, in a recent study we have shown that the delocalisation of *XIST* RNA and loss of H3K27me3 from the Xi are early events that precede partial Xi reactivation during cell fusion-mediated reprograming [68]. Interestingly, we show here that a second set of genes become susceptible to reprograming-mediated reactivation following DNA demethylation. These genes show high H3K9me3 and low H3K27me3 levels, suggesting that DNA methylation might be differentially regulated across the human Xi through proteins such as SMCHD1 that can bind H3K9me3 domains and mediate DNA methylation [69, 70].

Perhaps the most surprising result from our study is the finding that Xi gene reactivation can be predicted from detailed transcript analysis in single cell-derived clones ahead of reprograming. Evidence that human Xi genes that are sensitive to reprograming-mediated reactivation show stochastic Xi expression in hFs was derived both from RNA-seq analysis of hF clones and from single-cell RNA-FISH studies. It seems unlikely that stochastic expression reflects escape from either DNA methylation or facultative heterochromatin because these genes had similar levels of H3K27me3 and H3K9me3 in expressing and non-expressing clones, and 5azaC treatment was not able to induce Xi expression. Treatment with 5azaC did, however, reveal the reactivation of a different subset of Xi genes upon reprograming.

## Conclusions

Overall, these results have two important implications. First, they suggest that multiple different modes of silencing exist along the human Xi. Silencing of reprograming-reactivated genes might depend on the intrinsic expression probability of the gene and rely on stochastic transcriptional events that are stabilised upon pluripotent conversion. Consistent with this hypothesis, we noted that increases in the expression of X-reactivated genes upon cell fusion-mediated reprograming originated from both the Xa and Xi. Interestingly, stochasticity has been proposed to regulate Xi expression across the regions of the human X chromosome that have been most recently added during evolution (i.e. XAR) [22] and where most of the reprograming-reactivated candidate genes lie. Distinct Xi regions might instead be silenced by multiple epigenetic modifications that need to be erased to permit reactivation. This was highlighted by the discovery of a second cohort of Xi genes that were reactivated only after DNA demethylation. Second, our data suggest that Xi genes that will be successfully reactivated following pluripotent reprograming can be reliably predicted ahead of fusion by comparing allelic expression patterns in somatic cell clones. This result underscores the importance of understanding the underlying causes of intrinsic transcriptional variability and mosaicism that occur in normal female tissues. It also raises the interesting possibility that cell types showing maximal Xi-allelic variation might be the most suitable targets for human iPSC reprograming where reactivation of the X chromosome is desired. Given the high incidence of disease-associated genes residing on the X chromosome [71, 72], investigating XCI in single cells and their clonal derivatives may help to better design rational strategies to achieve locus-specific or domain-specific Xi reactivation.

## Methods

### Cell culture and fusion

Human fibroblasts NHDF17914 (Lonza) were immortalised with pBABE-hTERT-blast and single-cell clones were derived in 3% O_2_. In order to confirm the expression of a single X chromosome, clones were screened by *PDHA1* and *ATRX* RFLP analysis as previously described [33]. Mouse ESCs E14Tg2a HPRT-/ were transfected with pCAG-puro [73] to derive a stable clonal line.

For fusion experiments, cells were mixed in 1:1 ratio and treated in suspension with poly-ethylene-glycol (PEG 1500, Roche), as previously described [32]. Briefly, for RNA-seq experiments, 7 × 10^7^ hFs were harvested and fused to an equal number of mESCs by adding 2 mL of 50% PEG and stirring at 37 °C for 90 s. PEG was subsequently diluted with 4 volumes of KO-DMEM, resuspended in mESC medium (KO-DMEM supplemented with 10% FCS, 1% non-essential amino acids, 2 mM L-glutamine, 50 μM β-mercaptoethanol and 10 μg/mL penicillin/streptomycin) and plated onto gelatinised dishes at a density of 10^6^ cells per 140 mm dish. Heterokaryons and hybrids were cultured at 3% O_2_ in mESC media supplemented with LIF and selected after 12 h by adding puromycin (1.5 μg/mL) and HAT (20 μM hypoxantine, 0.08 mM aminopterin and 3.2 mM thymidine; Sigma). At each timepoint, 10^5^ cells were collected for RNA extraction. All experiments have been performed with hF clones between passages 8 and 14.

### Allele-specific Taqman PCR

Taqman PCR was performed with Taqman Universal Master Mix No AmpEraseUNG (Applied Biosystems) on RNAse-H treated single-strand cDNA (First Strand Synthesis kit, Invitrogen) or genomic DNA. Allele-specific Taqman probes (Applied Biosystem) were used for the analysis.

### RNA-sequencing

Total RNA was extracted using RNABee (Amsbio) and treated with 0.5 units/μg of DNAseI (Turbo DNAse kit; Ambion) to degrade traces of gDNA. Complete removal of gDNA contamination was tested by PCR amplification of total RNA and only samples with no amplicon upon 40 PCR cycles were processed. Libraries were prepared from 0.6 μg of total RNA (RIN >7.5) by True-seq RNA sample prep v2 kit (Illumina) and were amplified by 13 PCR cycles. Paired-end 100 bp reads were generated using HiSeq2500 sequencer (Illumina). GEO access number: GSE60308.

### Heterozygous SNP identification from RNA-seq

To identify heterozygous SNPs along the X chromosomes of NHDF17914 cells, we sequenced RNA libraries obtained from two clones with reciprocal XCI patterns (Strategy 1, Additional file 1: Figure S1). We mapped RNA-seq reads obtained from two biological replicates of each clone to the Human genome (hg19) using Tophat v2.0.8/Bowtie 2.1.0 [74, 75]. Biological replicates were merged following alignment. For each clone, we used SAMtools mpileup and BCFtools to separately identify the observed base (i.e. the most abundant base) and its phred scaled quality score at every genomic position along the X chromosome. We considered only positions with a minimum of eight overlapping reads and phred scaled quality score ≥20 in both clones. The positions at which a different base was identified as the most abundant in reciprocal clones were selected as putative heterozygous SNPs. We annotated the SNPs using Annovar [76], discarded SNPs overlapping more than one gene and retained only the SNPs within mature transcript regions (i.e. exons and UTR). The final dataset is composed of 379 heterozygous SNPs across 183 genes. Full list of the SNPs is reported in Additional file 2: Table S1.

An alternative/independent approach (Strategy2 in Additional file 1: Figure S1) was used to validate the identified SNPs. This second approach is based on the different assumption that gene expression levels will be almost equivalent in the two clones thus mirroring genomic ratios. Briefly, SAMtools was used for directly identifying heterozygosity (Hardy-Weinberg equilibrium hypothesis) from merged clone 12 and clone 34 alignments. This second strategy found 355 heterozygous SNPs out of which 354 were in common with previous analysis, thus showing the robustness of our approach.

### Human-specific alignments and allelic expression analysis of X-linked genes

RNA-seq was performed on hF clone 12 and 34 before and at 0, 4 and 6 days after mESC fusion in two independent fusion experiments per clone. To analyse the human-specific transcripts, RNA-seq reads were aligned independently to human (hg19, Ensembl gene version 72) and mouse reference genome (mm9, Ensembl gene version 67) using Tophat v2.0.8 and the reads aligning to both human and mouse genomes were excluded from further analysis. The number of SNP-overlapping reads for each allele was obtained using SAMtools mpileup.

In order to reconstruct the haplotypes of the two X chromosomes, at each SNP position the base with the highest read depth in hF clone 12 was considered as the Xa allele in this clone and the Xi one in clone 34. The Xa and Xi alleles were considered the same for all the samples in a time series.

To calculate allelic expression, we summed up the reads overlapping the Xa or the Xi alleles at the different SNPs along the same gene and excluded SNP positions overlapping two or more gene transcripts.

We used a beta-binomial model to assess the XCI status quantitatively and estimate the probability that a gene is inactivated. The beta-binomial distribution has been previously used to model differences in allelic expression [77] and estimate XCI [36, 78] by taking into account read count information (e.g. nucleotide variation and base sequencing quality) and overcoming the over dispersion of RNA-seq data. For each gene, we modelled the probability of inactivation as previously described [36]. Briefly, we estimated the total number of Xi-specific reads, the sum of base quality scores within these reads and the Xi ratio versus total; we fitted these data to a mixture of two beta-binomial distributions, one accounting for genes subject to inactivation and one for genes expressed from the Xi. When observing a probability of inactivation (τ_i0_) <0.05 we considered a gene significantly expressed from the Xi with 95% confidence.

In order to distinguish between genes that are reactivated (green) and the ones that are already active ahead of reprogramming (purple) or remain subject to XCI (red) across reprograming, we separately estimated the probability of inactivation (τ_i0_, see Additional file 4: Table S3) in hF and at 0, 4 and 6 days after mESC-fusion. To increase confidence in annotation, only genes with minimum 20 SNP-overlapping reads (in hF or day0 and in day4 and day6) were considered (87 genes) and classified as follows: ‘reactivated’ genes, which are not expressed from the Xi (τ_i0_ > 0.05) in hF/day0 but acquire significant Xi expression (τ_i0_ ≤ 0.05) at one data-point of the time series in at least one clone after reprograming (green); ‘active’ genes, which have significant Xi expression (τ_i0_ ≤ 0.05) in hF/day0 before reprograming and at one data-point of the timeseries after reprograming in at least one clone (purple); ‘inactive’ genes, which are not significantly expressed from the Xi (τ_i0_ > 0.05) in hF and all data-points of the time series in both clones (red). Genes with less than 20 SNP-overlapping reads across the time series were not evaluated.

Data obtained from two independent biological replicates for each clone were either merged by summing up Xi and Xa SNP-specific reads and their base quality scores (Additional file 4: Table S3) or analysed separately. Single replicates showed a good concordance both for Xi expression ratios (mean ± standard error is shown Fig. 3) and for beta-binomial statistics (concordance between replicates for SNPs with more than 10 overlapping reads was of 98%, 99%, 95% and 97% in clone 12 for hF, days 0, 4 and 6 respectively; and 94%, 97% and 89% in clone 34 for hF, days 0 and 4, respectively) with variations mainly due to different sequencing depth among replicates and not affecting the final classification compared to merged data.

### Gene expression analysis

We counted the reads overlapping with genes using HTseq and identified the differentially expressed genes using edgeR [79]. FPKM values were estimated using R script.

Reproducibility between independent biological replicates (i.e. separate fusion experiments), or between distinct clones, was assessed by Spearman correlation analysis using FPKMs and R script. Correlation coefficients (r) comparing gene expression between independent replicates were higher than 0.90 and 0.87 in clones 12 and 34, respectively. The same comparison between clones 12 and 34 gave r values ≥ 0.88 for all timepoints.

### RNA-FISH analysis and probes

Probes used for RNA-FISH analysis were obtained by nick translation of appropriate phosmids/BACs containing *HDAC8*, *TBLX1*, *CTPS2* and *RP11-706O15.1* (i.e. RP11-1021B19, RP11-451G24, CTD-2277I2 and WI2-1543 K8, respectively), in the presence of fluorophore-coupled dUTPs as previously described [80]. Locus-specific transcriptional signals were detected in hF nuclei and scored according to the detection of a single focus, of two separate foci (>1 microns apart) or >2 signals per nucleus [68]. More than 200 cells were scored in each sample.

## Additional files

Additional file 1: Supplementary figures and legends. (PDF 2566 kb)
Additional file 2: Table S1. Heterozygous SNP dataset. (XLSX 63 kb)
Additional file 3: Table S2. RNA-seq reads aligned to human (hg19) or to mouse (mm9) genomes. (XLSX 21 kb)
Additional file 4: Table S3. Allele-specific RNA-seq analysis of X-linked genes in hF clones 12 and 34 before and after mESC fusion. (XLSX 32 kb)
Additional file 5: Table S4. Allele-specific RNA-seq analysis of X-linked genes in hF clones 11, 12, 27 and 34 ahead of reprograming. (XLSX 34 kb)
