# Supplementary material for: Allele-specific analysis of cell fusion-mediated pluripotent reprograming reveals distinct and predictive susceptibilities of human X-linked genes to reactivation
Source: Genome Biol. 2017 Jan 25;18:2. doi: 10.1186/s13059-016-1136-4 (PMC5264468; doi:10.1186/s13059-016-1136-4)
Supplement: Additional file 1: — Supplementary figures and legends. (PDF 2566 kb) [file 13059_2016_1136_MOESM1_ESM.pdf]

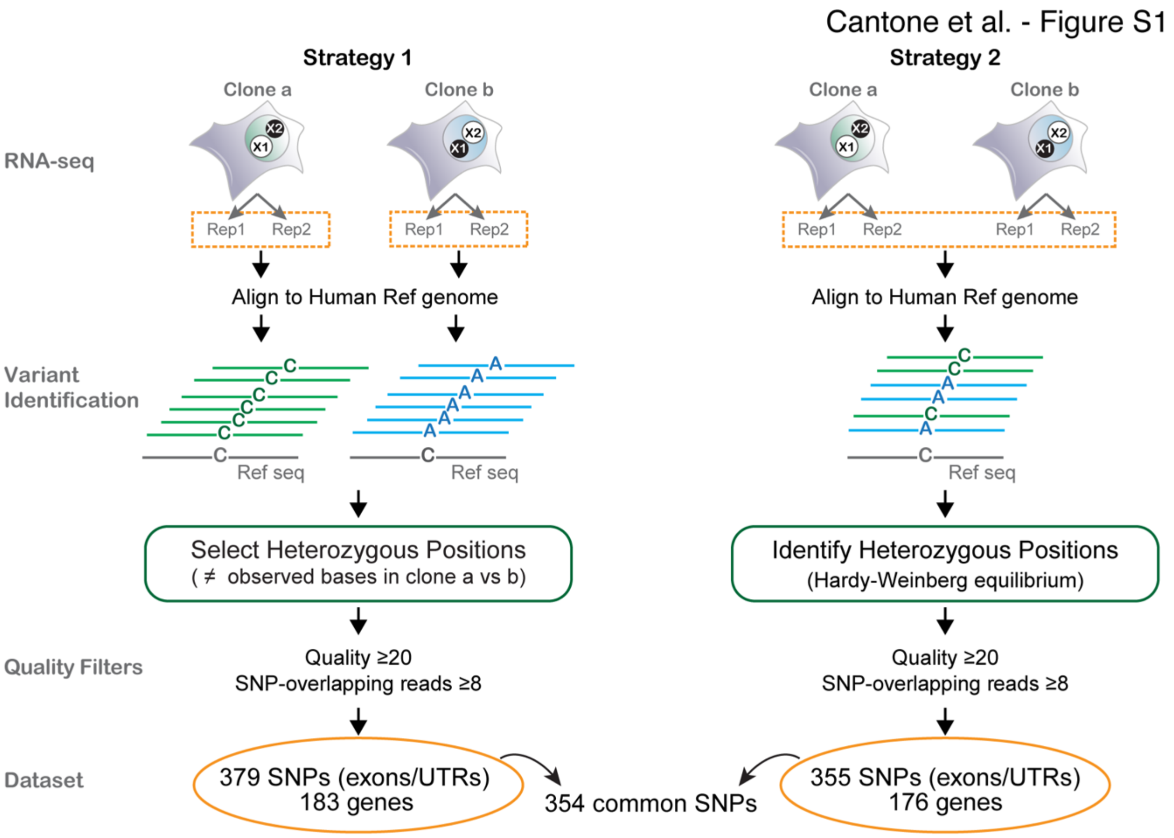

Figure S1. RNA-sequencing strategies to identify heterozygous SNPs. RNA-sequencing was performed using female hF clones in which reciprocal X chromosome pairs were active/inactive (e.g. X_1aX_2i and X_1iX_2a in clone types a and b, respectively). In the first strategy (strategy1) the reads from clone a (e.g. clone 11 and 12) and clone b (e.g. clone 27 and 34) were separately aligned to the reference genome sequence and those positions at which a different variant nucleotide was identified were considered heterozygous. In the second approach (strategy2) reads from both clones were merged before variant identification and processed as genomic DNA [1].

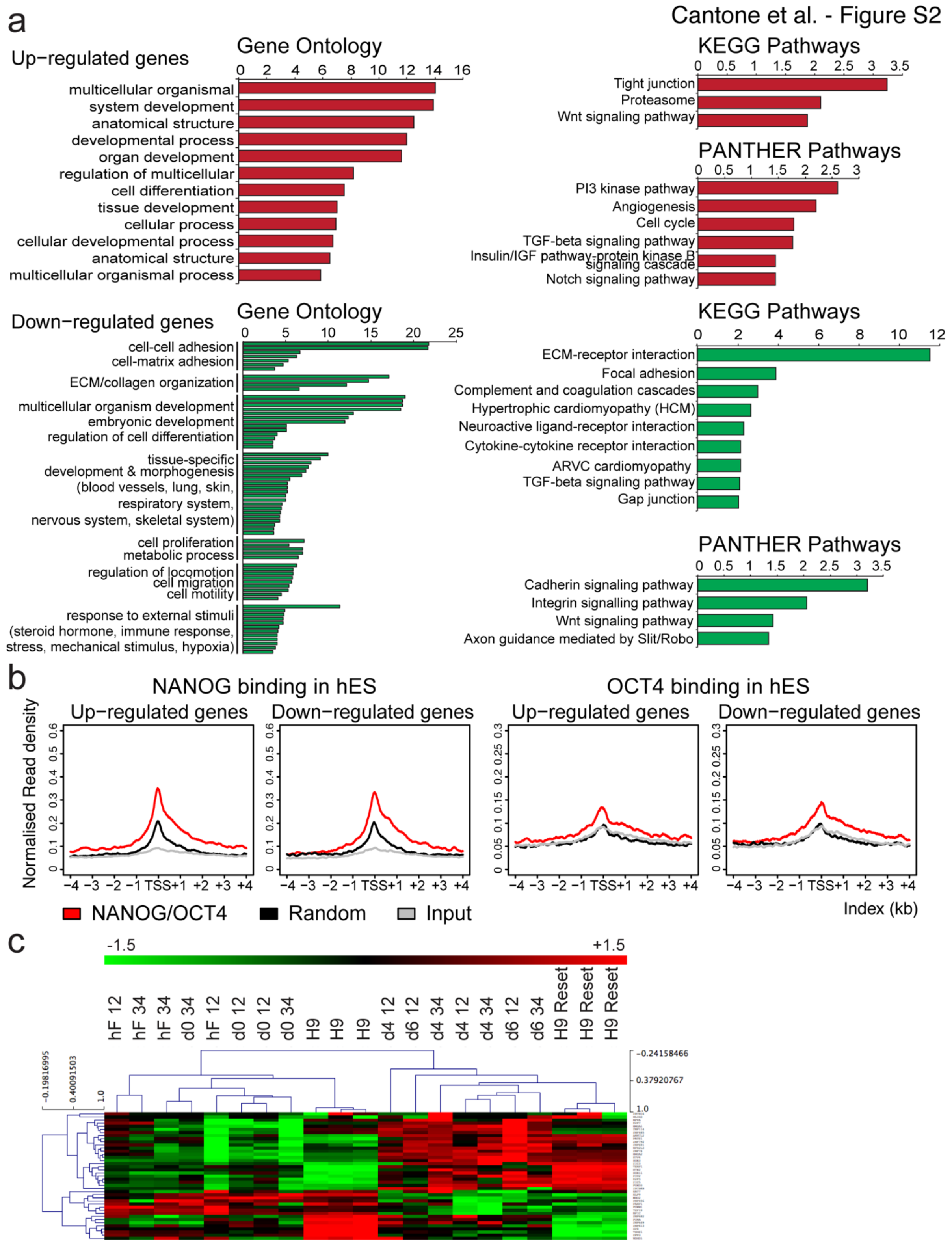

Figure S2. Induction of a human pluripotency gene program in hF clones upon fusion with mouse ESCs. (a) Functional enrichment analysis of Gene Ontology categories [2] for significantly up-regulated (red) and down-regulated (green) human genes in hFxmESC at day 4 and day6 versus day 0 (FDR <0.05; fold change ≥ 2). Graphs show the GO categories plotted against the p values (logarithmic

scale). **(b)** Enrichment of genes that are NANOG or OCT4 targets in human ESCs, and among genes that show differential expression in hFxmESC at days 4 and 6, versus day 0. Lines represent average OCT4 or NANOG immunoprecipitation (normalised read density in hESC) [3] within the set of differentially expressed genes (red), a random set of genes (black) or the input (grey). **(c)** Hierarchical clustering of genes that are up-regulated in hFxmESC but not in hESC (H9) and *vice versa* (black lines in figure 2b). H9 and H9 Reset represent primed and naïve hESC respectively, as described in [4]. Values correspond to the expression level (rlog, regularized logarithmic transformation) in each sample (hF clones 12 and 34; days 0, 4 and 6) scaled by the mean expression of each gene across samples.

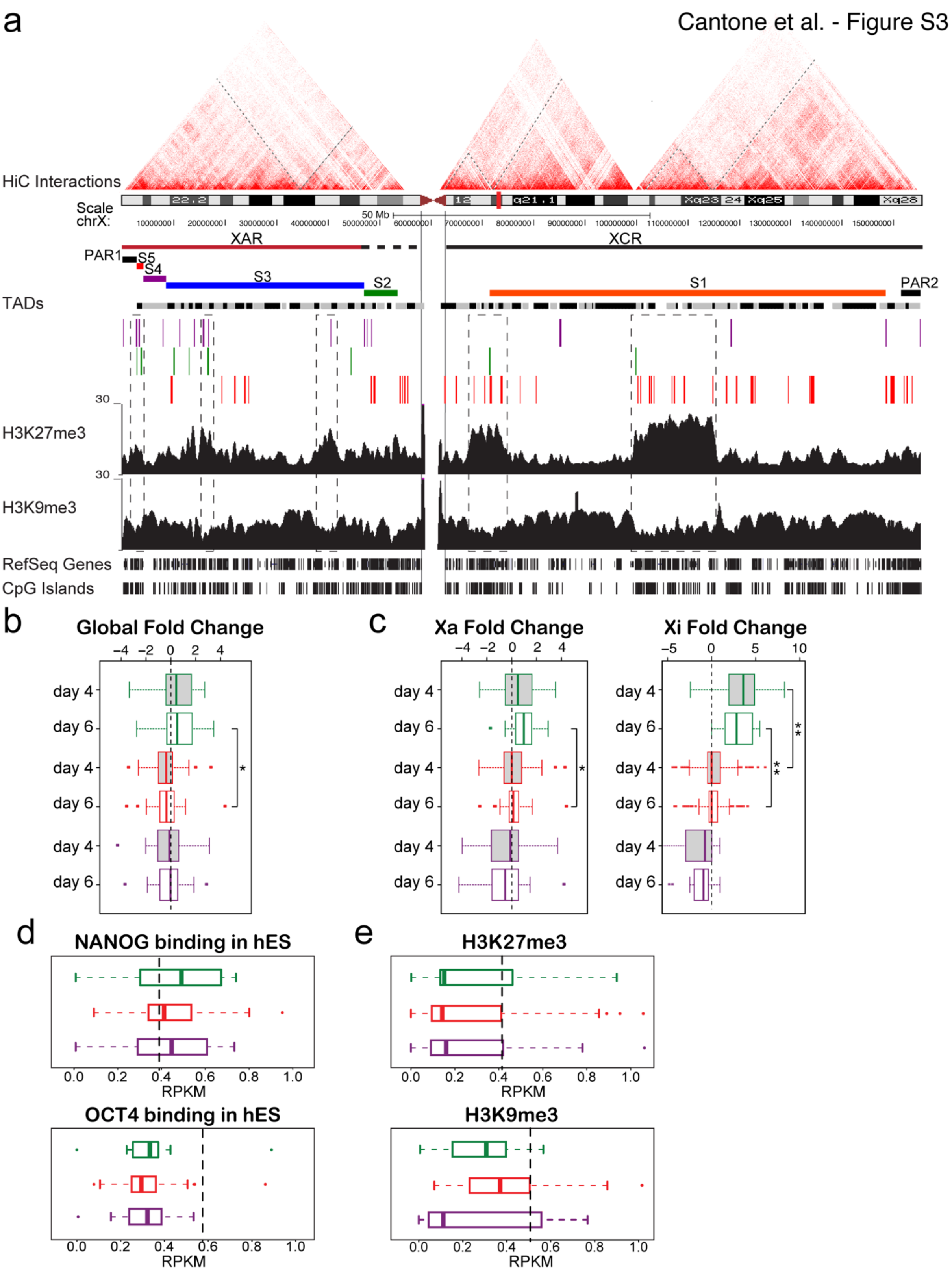

Figure S3. Influence of local chromatin environment on the reactivation of human Xi genes upon pluripotent reprogramming. (a) Schematic representation of the human X chromosome showing genetic and epigenetic features and the positions of genes that accordingly to RNA-seq in clone 12 and 34 have significant Xi expression across reprogramming (purple), are reactivated (green) or remain

inactive (red). TADs have been described previously [5] on the basis of HiC data. Regions showing different evolutionary conservation are represented as lines parallel to the X chromosome ideogram and are defined as in [6]: X Added Region (XAR), X Conserved Region (XCR), and evolutionary strata (PAR1, PAR2 and S1-5). H3K27me3 and H3K9me3 enrichment levels were defined using histone ChIP data from female human fibroblasts previously published in [7]. Reference gene sequences (RefSeq Genes) and CpG islands are obtained from the USCS Genome Browser. **(b)-(e)** Box plots showing reactivated (green), stably active (purple) or inactive (red) genes. **(b)** Fold change difference between transcript levels in hFxmESC at day 4 (grey-filled boxes) and day 6 (white-filled boxes) versus day 0. Fold change was calculated from our RNA-seq dataset by edgeR and represents the average of 3-4 independent experiments in clone 12 and clone 34. Dashed lines indicate zero change. Asterisks mark significant differences among gene categories (*) $p < 0{,}05$ and (**) $p < 0.0005$ (Mann-Whitney U test). **(c)** Xa- and Xi-specific fold change was computed using allele-specific reads and is represented as described in (b). **(d)** NANOG and OCT4 binding at gene transcriptional start sites (+/- 4kb) in male human ESC (H1) [3]. Dashed lines indicate mean X chromosome values. **(e)** H3K9me3 and H3K27me3 enrichment along the gene body. Data are represented as reads per kilobase per million (RPKM) obtained from published data of human fibroblasts [7]. Dashed lines indicate mean X chromosome values.

Cantone et al. - Figure S4

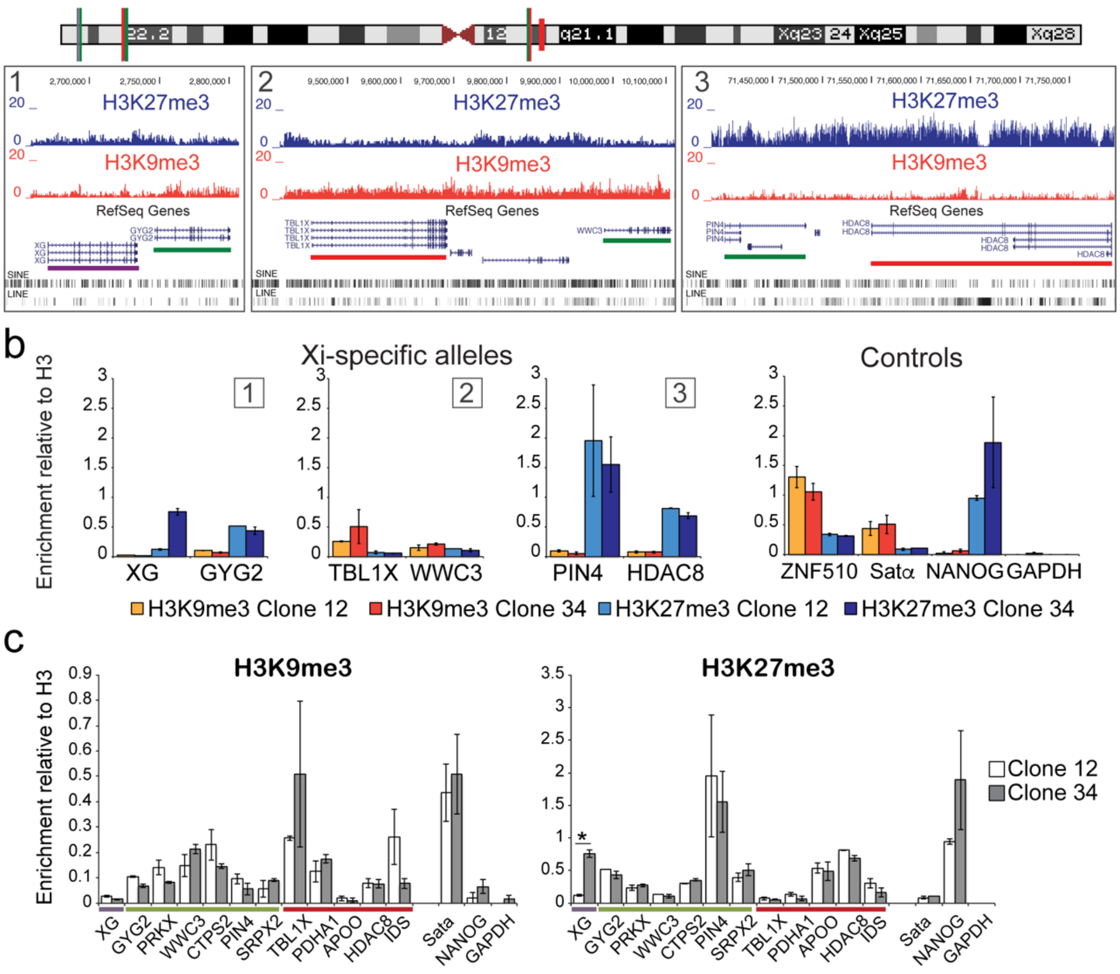

Figure S4. Reactivated genes have similar H3K9me3 and H3K27me3 in expressing and non-expressing clones. (a) Enrichment of H3K27me3 (blue) and H3K9me3 (orange) at three distinct X chromosome TADs (marked) neighboring genes with different status upon reprogramming: reactivated (green), active (purple) or inactive (red). **(b)-(c)** Xi-specific enrichment of H3K27me3 and H3K9me3 in hF clone 12 and 34. Data represent enrichment relative to H3 immunoprecipitation.

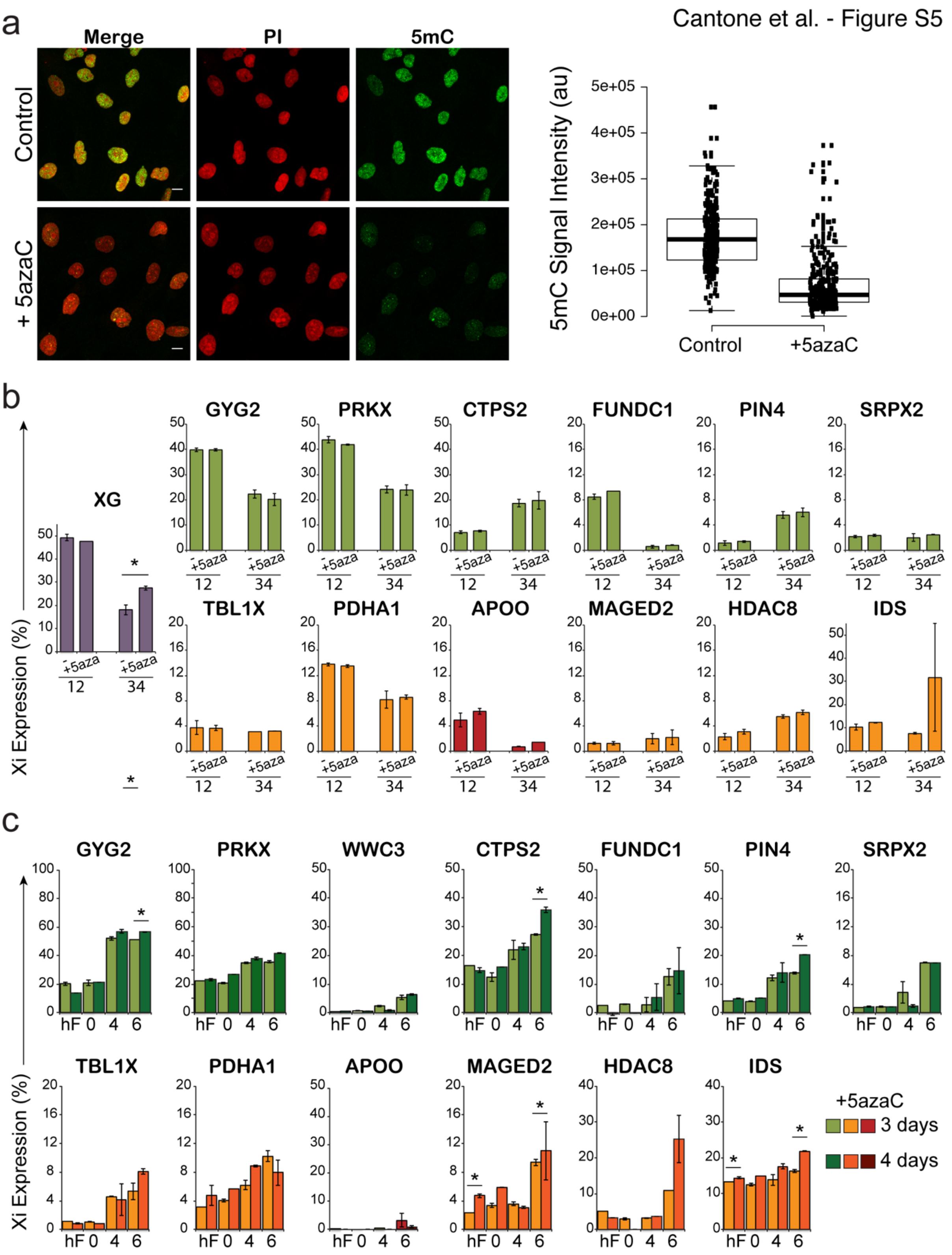

Figure S5. DNA demethylation does not induce reactivation of stochastically expressed Xi genes ahead of reprogramming. (a) Confocal images show 5-methyl-cytosine (5mC) immunofluorescence and DNA staining (Propidium Iodide, PI) of human fibroblasts that were cultured for 3 days in the presence of 1μM 5-deoxy-azacytidine (+5azaC) or were untreated (control). Box plots on the right show

5mC intensity density across nuclei of 5azaC treated (n=302) and control (n=254) cells. **(b)** Xi gene expression in hF clone 12 and 34 after culture for 3 days in the presence (+5azaC) or absence of 5-deoxy-azacytidine. Data represent the average of 2 independent experiments ± SEM for each clone. Asterisks (*) marks significant differences ($p \leq 0.05$, two-sided t-test). **(c)** Xi allelic expression in hF clone 34 treated for 3 or 4 days with 5azaC and then reprogrammed by fusion with mESCs. Data represent the average of 2 independent experiments ± SEM for each clone. Asterisks (*) marks significant differences ($p \leq 0.05$, two-sided t-test).

Supplemental References

1. Harvey CT, Moyerbrailean GA, Davis GO, Wen X, Luca F, Pique-Regi R: **QuASAR: quantitative allele-specific analysis of reads.** *Bioinformatics* 2015, **31:**1235-1242.

2. Huang da W, Sherman BT, Lempicki RA: **Systematic and integrative analysis of large gene lists using DAVID bioinformatics resources.** *Nat Protoc* 2009, **4:**44-57.

3. Gerstein MB, Kundaje A, Hariharan M, Landt SG, Yan KK, Cheng C, Mu XJ, Khurana E, Rozowsky J, Alexander R, et al: **Architecture of the human regulatory network derived from ENCODE data.** *Nature* 2012, **489:**91-100.

4. Takashima Y, Guo G, Loos R, Nichols J, Ficz G, Krueger F, Oxley D, Santos F, Clarke J, Mansfield W, et al: **Resetting transcription factor control circuitry toward ground-state pluripotency in human.** *Cell* 2014, **158:**1254-1269.

5. Dixon JR, Selvaraj S, Yue F, Kim A, Li Y, Shen Y, Hu M, Liu JS, Ren B: **Topological domains in mammalian genomes identified by analysis of chromatin interactions.** *Nature* 2012, **485:**376-380.

6. Kelkar A, Thakur V, Ramaswamy R, Deobagkar D: **Characterisation of inactivation domains and evolutionary strata in human X chromosome through Markov segmentation.** *PLoS One* 2009, **4:**e7885.

7. Nozawa RS, Nagao K, Igami KT, Shibata S, Shirai N, Nozaki N, Sado T, Kimura H, Obuse C: **Human inactive X chromosome is compacted through a PRC2-independent SMCHD1-HBiX1 pathway.** *Nat Struct Mol Biol* 2013, **20:**566-573.
